# Volitional control of saccadic adaptation

**DOI:** 10.1371/journal.pone.0210020

**Published:** 2019-01-10

**Authors:** Frauke Heins, Annegret Meermeier, Markus Lappe

**Affiliations:** 1 Department of Psychology, University of Muenster, Muenster, Germany; 2 Otto Creutzfeldt Center for Cognitive and Behavioral Neuroscience, University of Muenster, Muenster, Germany; Monash University, AUSTRALIA

## Abstract

Saccadic adaptation is assumed to be driven by an unconscious and automatic mechanism. We wondered if the adaptation process is accessible to volitional control, specifically whether any change in saccade gain can be inhibited. Participants were exposed to post-saccadic error by using the double-step paradigm in which a target is presented in a peripheral location and then stepped during the saccade to another location. In one condition, participants were instructed to follow the target step and look at the final target location. In the other condition they were instructed to inhibit the adjustment of saccade amplitude and look at the initial target location. We conducted two experiments, which differed in the size of the intra-saccadic target step. We found that when told to inhibit amplitude adjustment, gain change was close to zero for outward steps, but some adaptation remained for inward steps. Saccadic latency was not affected by the instruction type for inward steps, but when the target was stepped outward, latencies were longer in the inhibition than in the adaptation condition. The results show that volitional control can be exerted on saccadic adaptation. We suggest that volitional control affects the remapping of the target, thus having a larger impact on outward adaptation.

## Introduction

Saccades are rapid ocular movements that align the fovea with objects of interest and are essential for the perception of our visual environment. Since saccades are so rapid, they cannot be guided by visual feedback obtained during their execution. This implies that the corresponding motor command has to be planned in advance to perform an accurate eye movement towards a target. Although the oculomotor system is subject to variations in muscle conditions such as fatigue or aging, the saccades mostly remain accurate.

To achieve this accuracy some form of motor learning has to be involved in the planning and execution of saccades. This motor learning is called saccadic adaptation and it occurs naturally, for example when extraocular muscles are weakened and thus the eye is constantly missing the target position [1,2]. Saccadic adaptation can also be induced by experimental manipulation, such as the double-step paradigm [3]. Through shifting the saccade target repeatedly over a series of trials while the saccade is still in flight, a post-saccadic error signal is evoked, which leads to a gradual adjustment of saccadic amplitude.

A post-saccadic error typically evokes a corrective secondary saccade. Since the execution of corrective saccades is not necessary for adaptation to occur [4,5], the error signal is assumed to be visual rather than motoric. Furthermore, the error signal seems to reflect the discrepancy between predicted and actual retinal image after saccade execution rather than the post-saccadic eccentricity of the target [6,7,8]. This finding could also be linked to the observation that many saccades are hypometric which shows an undershoot bias of the saccadic system [9]. The undershoot bias is actively maintained after changes in the visual feedback [10,8], which supports the idea that the oculomotor system prefers hypometria over hypermetria.

The gain decrease following inward target steps seems to rely more on internal motor adjustments, while increase of gain following outward target steps requires target remapping [11,12,13]. In addition to that, a difference in the time course of adaptation between inward and outward adaptation has been noted: inward adaptation usually is more complete than outward adaptation and is obtained faster [14,15,16]. Similarly, recovery from outward adaptation requires fewer trials than recovery of inward adaptation. Indeed, de-adaptation from inward adaptation necessitates more trials than the preceding adaptation process [17], suggesting that the recovery from gain decrease is like outward adaptation, which has a longer time course than inward adaptation.

The slow and gradual changes of amplitude during adaptation and also during de-adaptation have been seen as evidence that saccadic adaptation is an involuntary process. Recovery from inward adaptation necessitates as many trials as the adaptation process itself, and recovery from outward adaptation still needs two-thirds of the trials required for the preceding adaptation of the amplitude [18]. Moreover, most participants that undergo the double-step procedure report no awareness of the intra-saccadic target-step when interviewed afterwards [3,19]. This is due to saccadic suppression of displacement [20], and may make any conscious alteration of behavior difficult. Yet, even experienced participants, who sometimes noticed the target displacement, showed no difference in the time course or the amount of adaptation in comparison to naïve participants [21]. Other research has pointed towards a strategic component in addition to the gradual learning process. Colleagues [22,15] investigated whether the amount of gain change achieved during double-step trials carries over to trials in which the target is simply removed during the saccade. They found that this is only partially the case, suggesting that during the confrontation with an intra-saccadic target shift some kind of strategic component might be active. This is in accordance with findings of our colleagues [23] who instructed participants to saccade very accurately to stationary, i.e. non-stepping, targets and to inhibit any corrective saccades. Not only did the participants execute very precise eye movements, they were also able to reduce the amount of corrective secondary saccades significantly. Despite these hints in favor of an existence of a voluntary component in the processing of post-saccadic error mediation, it is not expected to be the dominant mechanism of adaptation, since the difference in amplitude between the end of the adaptation phase and target-removed-trials is rather small.

In the present study we conducted two experiments to examine the ability to inhibit saccadic adaptation voluntarily. In both experiments we used the common double-step paradigm [3] with different instructions. In a total of four different conditions, participants were either instructed to fixate the fixation cross and then, upon presentation of the first target, to perform a saccade to that position and remain fixated at that position, independent of any possible further movement of the target, or to continuously follow the target to its final position. These instructions were given for both inward and outward target displacement.

## Experiment 1

### Method

#### Sample

The sample consisted of eight participants (3 male, 5 female) aged between 17 and 53 years (M = 29, SD = 11.25). All participants were right handed and had normal or corrected-to-normal vision. The participants were recruited from the Department of Psychology and gave informed consent concerning the collection and analysis of the data in written form. Except for one, all participants were experienced with oculomotor testing.

#### Experimental setup

Experimental procedures followed the Declaration of Helsinki and were approved by the Ethics Committee of the Department of Psychology and Sports Science of the University of Muenster. The experiment was conducted in a dimly lit room in the Institute of Psychology of the University of Muenster. The participants were seated 57 cm in front of an Eizo FlexScan 22-inch monitor (Eizo, Hakusan, Japan). The visual display size was 40 x 30 deg and the screen resolution 1152 x 864 pixel at an image refresh rate of 75 Hz. The stimuli were presented on a mid-grey background. A fixation cross (0.6 x 0.6 deg) was displayed close to the center of the screen. The position of the fixation cross varied by 2 deg on the vertical axis (up to 1 deg upward or downward), and 4 deg on the horizontal axis (up to 2 deg to the right or left) in a counterbalanced manner between trials. Eye positions were measured at a sampling frequency of 1000 Hz using an Eyelink 1000 eye tracking system (SR Research, Ontario, Canada). When a stable eye-position within a 3 deg range from the fixation cross was detected, and a random timespan between 700 and 1500 ms had elapsed, the fixation cross was removed and the target appeared. The target was a dark grey circle of 0.5 deg diameter. It appeared 12 deg to the right of the fixation cross. The eye position was continuously monitored and the intra-saccadic step in the double-step trials, or the elimination of the target in the target-off trials, was elicited when the eye had moved by 3 deg toward the target. The experiment was run in MATLAB R2014b (Mathworks, Natick, MA) with the Psychophysics Toolbox [24]. Viewing was binocular, but only the left eye was recorded. A chin rest was used to ensure a stable head position during the recording session.

#### Experimental design

The experiment consisted of four recording sessions per participant, one for each of the four adaptation conditions we measured. The inhibition and the adaptation conditions were measured for both inward and outward target steps, resulting in the experimental conditions inward inhibition, inward adaptation, outward inhibition and outward adaptation. For the purpose of reducing adaptation carry-over from the preceding recording session, the minimum time interval between two recording sessions was set at 72 hours and all participants completed the two inhibition conditions first before participating in the adaptation conditions. The order of inward and outward conditions was counterbalanced between participants.

Each of the four experimental conditions consisted of 240 trials and began with 20 pre-adaptation trials. Those were followed by 150 double-step adaptation trials and subsequent 70 post-adaptation trials. Every 50 trials the experiment paused for 25 s and an audio file was being played, containing an oral instruction for the respective condition and thus reminding the participant of the task.

In the pre-adaptation trials and the post-adaptation trials the participants first had to look at the fixation cross and then saccade toward the target. The target disappeared during the saccade.

**Fig 1:**
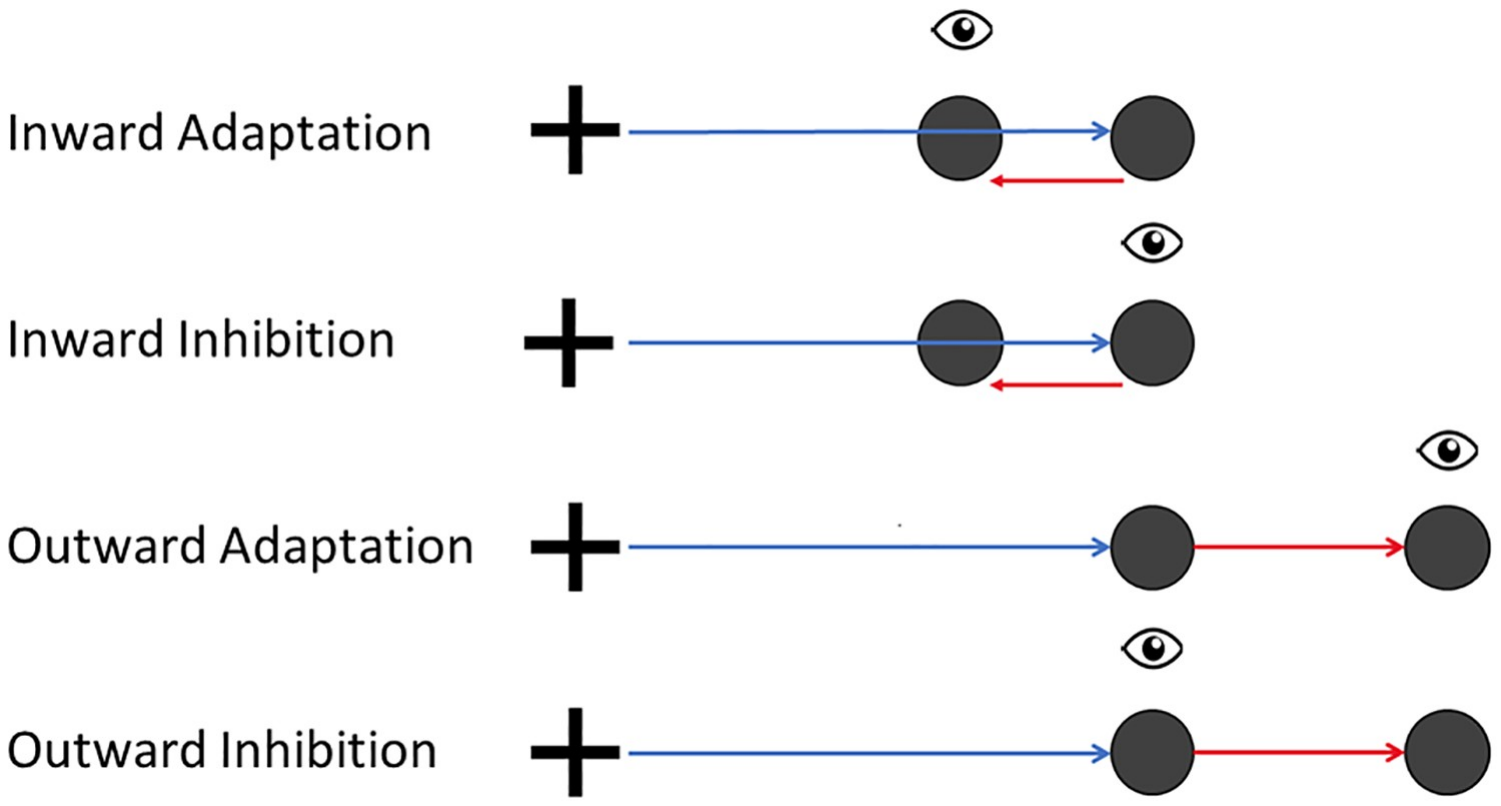
The double-step paradigm for inward and outward target displacement. In the inward as well as in the outward conditions the target appears at the initial target position T1, 12 deg to the right of the fixation cross. On saccade detection toward T1, the target is shifted to position T2. In the inward conditions T2 is displayed left from the first landing point, while in the outward conditions the target is stepped to the right.

The experimental manipulation took place in the adaptation trials. To induce saccadic adaptation, the double-step paradigm [3] was used. As soon as the participant initiated the saccade to the target position, the target stepped again while the saccade was still in flight. This intra-saccadic target step resulted in a post-saccadic visual error, hence evoking corrective saccades to the new target location. After repeated performance of double-step trials, the amplitude of the initial saccade was expected to become longer or shorter. Fig 1 shows the double-step paradigm for inward as well as outward target displacement.

The size of the intra-saccadic target step was 20, 30 or 40 percent of the original saccade amplitude (12 deg), hence the absolute size of the intra-saccadic target displacement was 2.4, 3.6 or 4.8 deg. These different step sizes were randomly intermixed during the series of adaptation trials to prevent the participants from predicting the target step in each trial and aiming their saccade at a fixed distance from the primary target. Intermixing different step sizes should not be a problem for the experiment since saccadic adaptation is possible when the step size is variable [25]. The target displacement was initiated as soon as the online detection algorithm detected the saccade, which happened after the position of the eye was shifted more than 3 deg from the fixation cross toward the target and the eye velocity exceeded 120 deg/s.

#### Instructions

Our study was aimed to test the ability to inhibit a hypothetic automatic process, namely saccade adaptation, which occurs in response to an intra-saccadic target step. Therefore, unlike in other experiments on saccadic adaptation, participants needed to be aware that the target could step multiple times in order to be able to understand the task. Hence, in the inhibition condition, they were instructed to fixate the fixation cross and upon presentation of the target, perform a saccade to that position and continue to look at that position even if the target performed any further movement. While they were not explicitly told that the target would step during the saccade on every trial of the adaptation phase, the possibility of further target movement after the initial step had to be indicated to the participant.

We used a comparable instruction for the adaptation task, since we wanted to vary only the task, not the description of the stimuli. Participants in the adaptation condition were told to look at the target and follow the targets’ movement to its final position.

The adaptation condition was measured after the inhibition condition in order to avoid that the inhibition condition was affected by any prior adaptation. Thus, at the time of the adaptation condition participants had already heard the inhibition instruction and knew the stimuli. However, it has previously been shown that participants who were aware of the intra-saccadic target step exhibited the same amount of adaptation as unaware participants [21].

#### Analysis

For the purpose of comparing the influence of the experimental manipulation on saccadic adaptation, the saccadic gain change was calculated and averaged per condition and participant. The gain change was calculated as following:

$$\text{GC}=\frac{A-{\overset{¯}{A}}_{pre}}{{\overset{¯}{A}}_{pre}}\times 100.$$

The average amplitude of the first 20 trials was subtracted from the amplitude of the respective trial and the resulting difference then was divided by the average amplitude of the first 20 trials. In order to display results in percent, the result was multiplied by 100. The differences in saccadic gain change then were investigated through a 2 x 2 repeated measures ANOVA. Post-hoc tests were performed to test for differences in the saccade characteristics between the different experimental conditions. Primary saccades with an amplitude greater than 18 deg or smaller than 6 deg, thus having over- or undershot the target by half of the required saccade size, were excluded from data analysis. The same applied to saccades with latencies of less than 100 ms and more than 400 ms, or to saccades which reached peak velocities of less than 100 or more than 900 deg/s. This affected 5.9% of all saccades in the first experiment and 3.7% in the second experiment.

In addition to gain change, the number of corrective saccades and the size of secondary saccades in the double-step trials were also investigated. To avoid the inclusion of saccades that were meant to guide the fovea back to the fixation cross, secondary saccades with an amplitude of more than 6 deg were excluded. If not mentioned differently, all t-tests were paired and had an alpha-level of .05. The post-hoc tests were Bonferroni-Holm-adjusted for multiple comparisons.

### Results

#### Gain change

**Fig 2:**
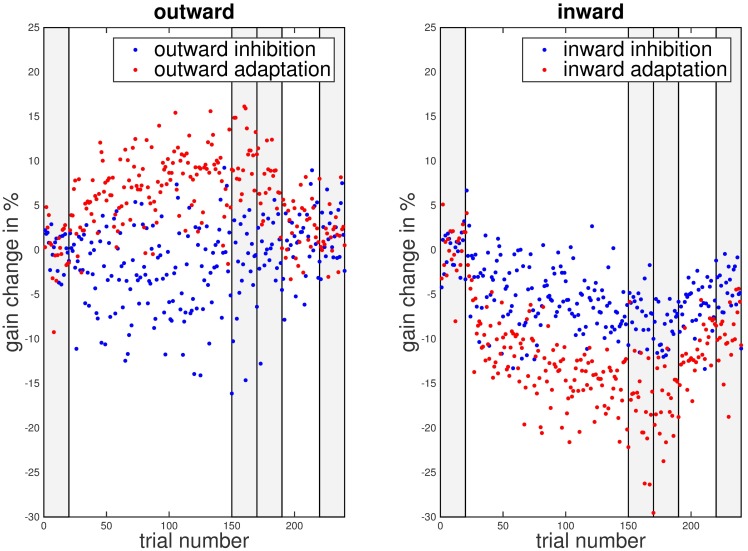
Gain change over trial number for the different instruction types and adaptation directions. Data from the inhibition conditions is marked by blue dots, data from the adaptation condition by red dots. The shaded areas mark the phases used for gain change analysis: pre-adaptation (trials 1–20), late adaptation (trials 151–170), post-adaptation (trials 171–190) and late post-adaptation (trials 221–240).

We wanted to investigate if the instruction to remain on the first landing position in the double-step paradigm prevented saccadic adaptation. Fig 2 illustrates the time course of saccadic gain changes over the adaptation phase. Each data point represents the average gain change for that particular trial across all participants. In the outward adaptation condition gain change followed the typical exponential learning curve. In the outward inhibition condition, in contrast, gain change remained around zero throughout the experiment. In the inward conditions, gain decreased in both conditions with an exponential time course but the gain change was much weaker in the inhibition than in the adaptation condition. Hence, both inhibition conditions showed weaker adaptation than the respective adaptation conditions, while some adaptation nonetheless occurred in the inward inhibition condition.

**Fig 3:**
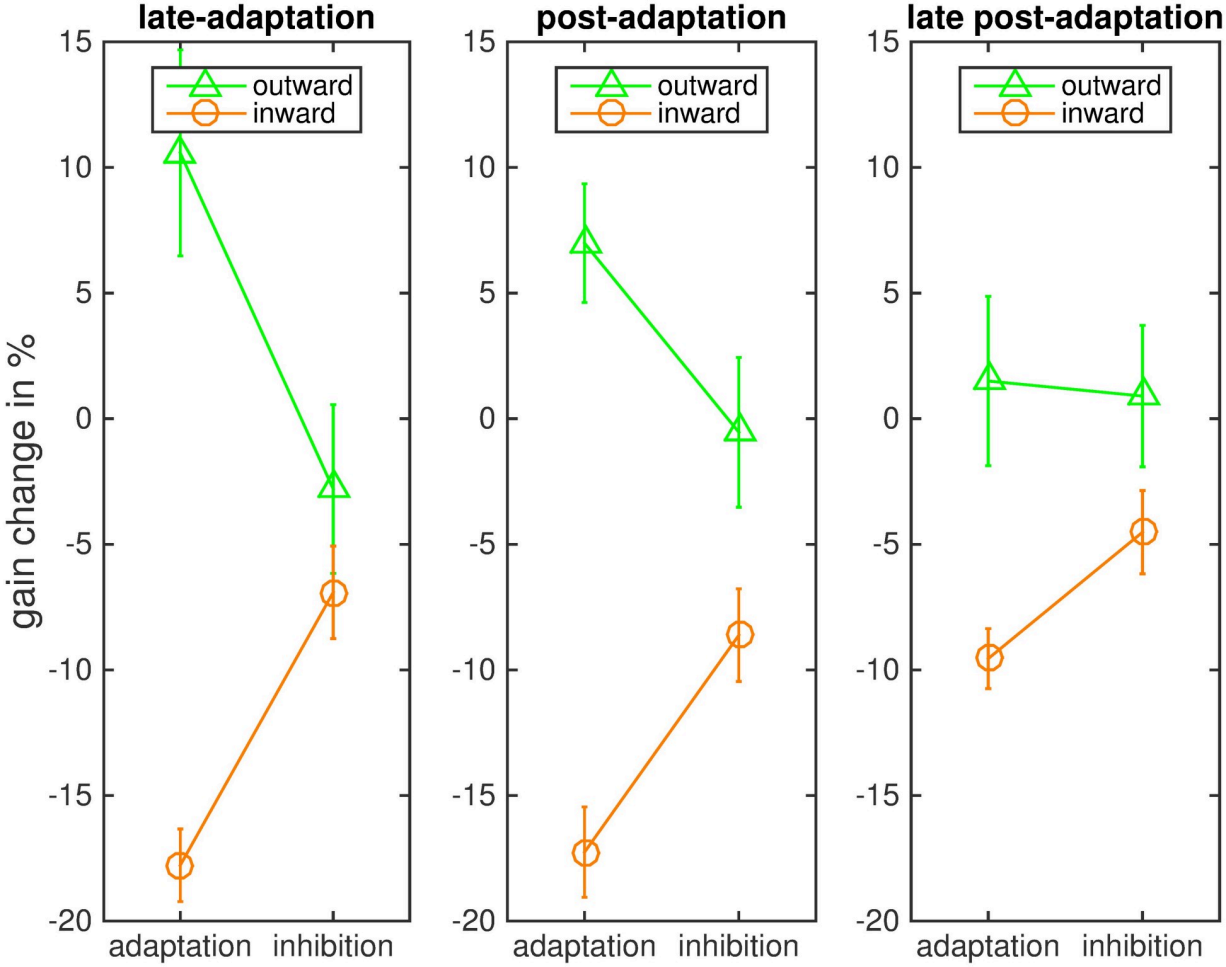
Mean saccadic gain change in the four conditions for each of the three phases. The bars indicate the standard error of the mean.

These effects were confirmed by the statistical analysis of mean saccadic gain change. We conducted our analyses for the last 20 trials of the adaptation phase, the first 20 post-adaptation trials and the last 20 post-adaptation trials (Fig 3). A 2 x 2 repeated measures ANOVA of the last-adaptation trials with the within-subject factors instruction and adaptation direction reported a significant main effect of the adaptation direction (F(1,7) = 23.389, p = .002, η^2^ = .770), which was expected as gain change was in opposite directions in the inward and outward conditions. There was no main effect of instruction (F(1,7) = 0.276, p = .616), which was also expected since gain change difference between the inhibition and the adaptation conditions was in opposite directions. The interaction of instruction and adaptation condition was of most interest and was significant (F(1,7) = 27.070, p = .001, η2 = .795). An ANOVA of the post-adaptation trials showed the same pattern. The main effect of adaptation direction was significant (F(1,7) = 62.406, p < .001, η2 = .899). There was no main effect of the instruction (F(1,7) = 0.045, p = .837) and the interaction of instruction and adaptation direction was significant (F(1,7) = 16.128, p = .005, η2 = .697). An ANOVA of the last 20 post-adaptation trials again showed the same effects: a significant main effect of adaptation direction (F(1,7) = 11.618, p = .011, η2 = .624), a significant interaction of adaptation direction and instruction (F(1,7) = 8.105, p = .025, η2 = .537) and no main effect of instruction (F(1,7) = 0.861, p = .384).

The mean gain change (and standard deviation) in the late adaptation trials was 10.58% (11.60%) in the outward adaptation condition, -2.80% (9.49%) in the outward inhibition condition, -17.78% (4.1%) in in the inward adaptation condition and -6.91% (5.21%) in the inward inhibition condition. For the post-adaptation trials mean gain change was 6.9% (8.5%) in the outward adaptation condition, -0.5% (10.7%) in the outward inhibition condition, -17.3% (7.4%) in the inward adaptation condition and -8.6% (6.7%) in the inward inhibition condition. For the last post-adaptation trials gain change was 1.50% (9.53%) in the outward adaptation, 0.9% (7.96%) in the outward inhibition, -9.55% (3.38%) in the inward adaptation and -4.52% (4.68%) in the inward inhibition condition. In the late-adaptation trials, gain change deviated from zero in the inward adaptation (t = -12.308, p < .001), the inward inhibition (t = −3.752, p = .007) and the outward adaptation condition (t = 2.581, p = .036), but not in the outward inhibition condition (t = -0.834, p = 0.432). The same pattern of results was found for the post-adaptation trials: Gain change deviated from zero in the inward adaptation (t = 9.600, p < .001), the inward inhibition (t = -4.672, p = .007) and the outward adaptation condition (t = 2.956, p = .042), but not in the outward inhibition condition (t = -0.181, p = .862). For the late post-adaptation trials, the gain change was significantly different from zero in the inward adaptation (t = -7.993, p < .001) and the inward inhibition condition (t = -2.728, p = .029). However, in both outward adaptation (t = 0.445, p = .670) and outward inhibition (t = 0.320, p = .758) gain change did not differ from zero.

#### Secondary saccades

**Fig 4:**
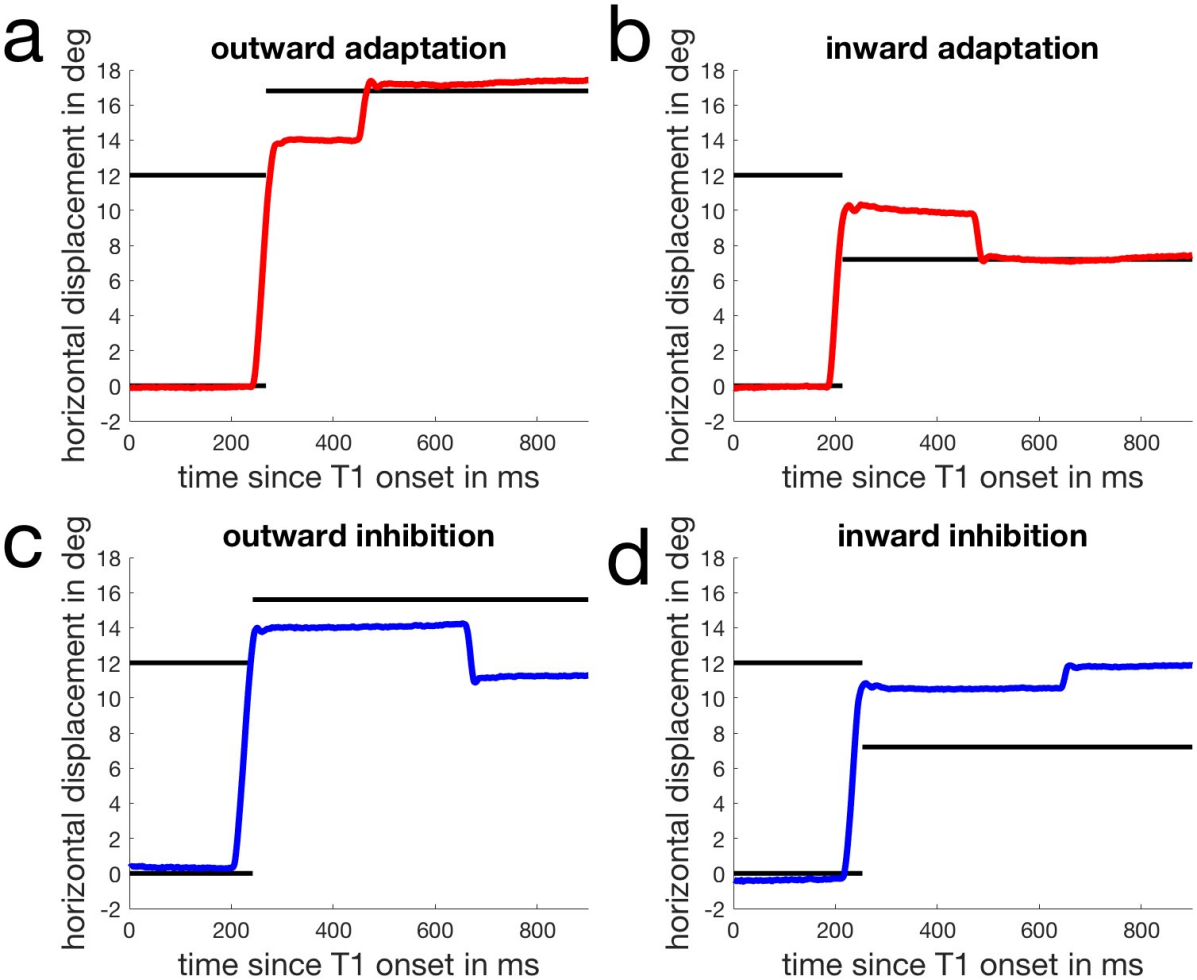
Examples of secondary saccades in the four conditions. a: In this outward adaptation trial the primary saccade is directed between the initial and the final target position and a secondary saccade is directed towards the final target position. This secondary saccade is a corrective saccade since it corrects for the visual error induced by the intra-saccadic target step. b: A similar corrective secondary saccade is directed towards the final target position in this inward adaptation trial. c: Example of a secondary saccade in the outward inhibition condition. After the primary saccade is made towards the initial target, the secondary saccade is directed towards the initial target position. d: A similar example from an inward inhibition trial.

In normal adaptation, the intra-saccadic target step induces corrective secondary saccades towards the final target position (Fig 4a and 4b).

**Fig 5:**
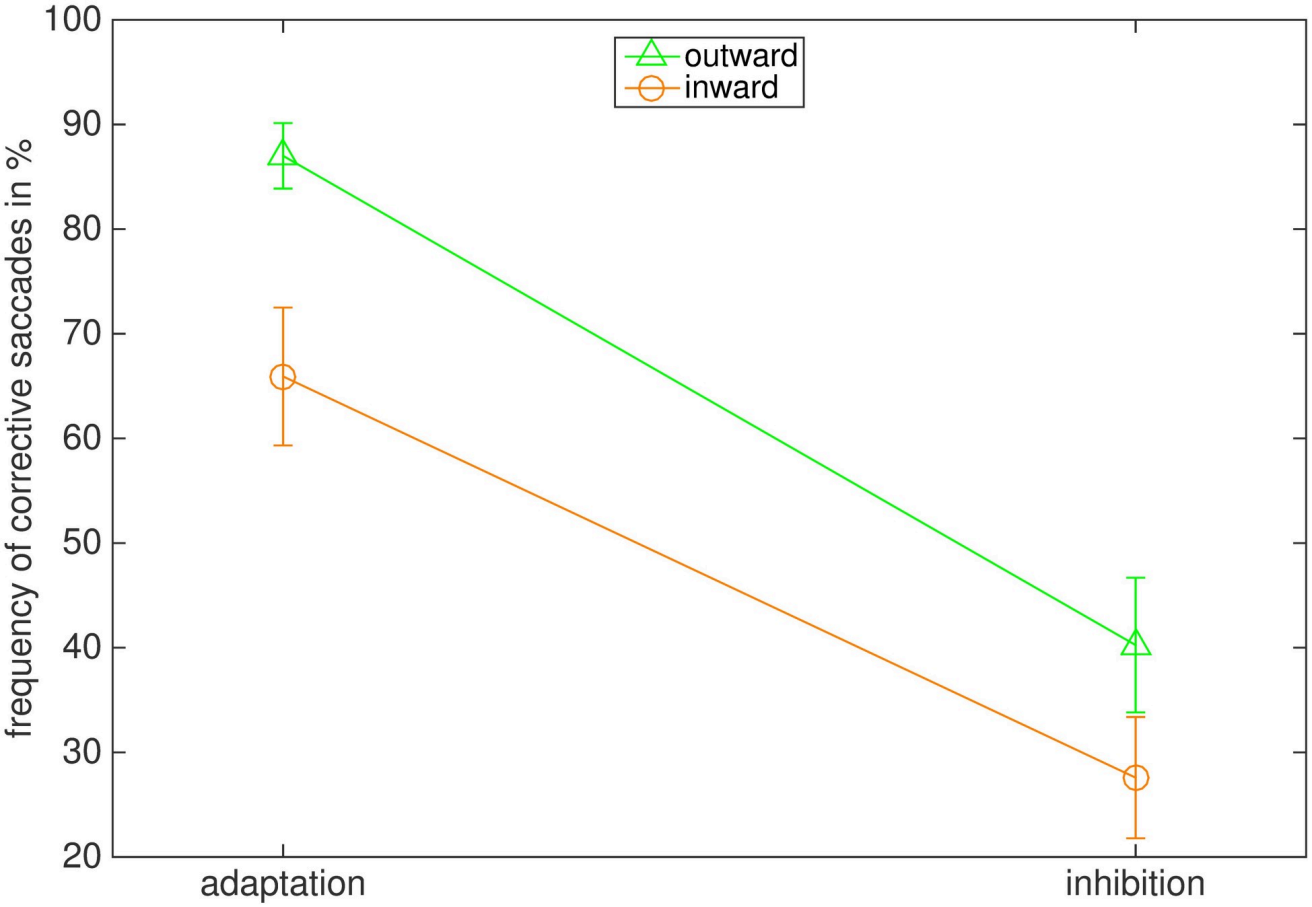
Average frequency of corrective saccades in the four conditions. The bars represent the standard error of the mean.

Fig 5 illustrates the effect of instruction and adaptation direction on the frequency of corrective saccades to the final target position. In both adaptation conditions more corrective saccades were executed than in the respective inhibition conditions, and more corrective saccades were executed in the outward than in the inward condition. The frequency of corrective saccades was 40.3% (18.2%) for outward inhibition and 87.0% (8.85%) for outward adaptation. For inward inhibition the frequency of corrective saccades was 27.6% (16.40%) and 65.9% (18.63%) for inward adaptation. The main effects of instruction (F(1,7) = 102.535, p < .001, η2 = .936) and adaptation direction (F(1,7) = 7.319, p = .030, η2 = .511) were confirmed by the 2 x 2 repeated measures ANOVA. The interaction of adaptation direction and instruction (F(1,7) = 0.345, p = .571) was not significant.

To assess whether the frequency of corrective saccades decreased across the experiment, we calculated GLMs with the predictor trial number for each condition. The frequency of corrective saccades decreased in the inward adaptation condition (ß^ = -0.011, t = -4.510, p < .001). For outward inhibition (ß^ = -0.002, t = -0.837, p = .404), outward adaptation (ß^ = -0.004, t = -1.847, p = .067) and inward inhibition (ß^ = 0.004, t = 1.379, p = .170) trial number had no influence on the frequency of corrective saccades.

**Fig 6:**
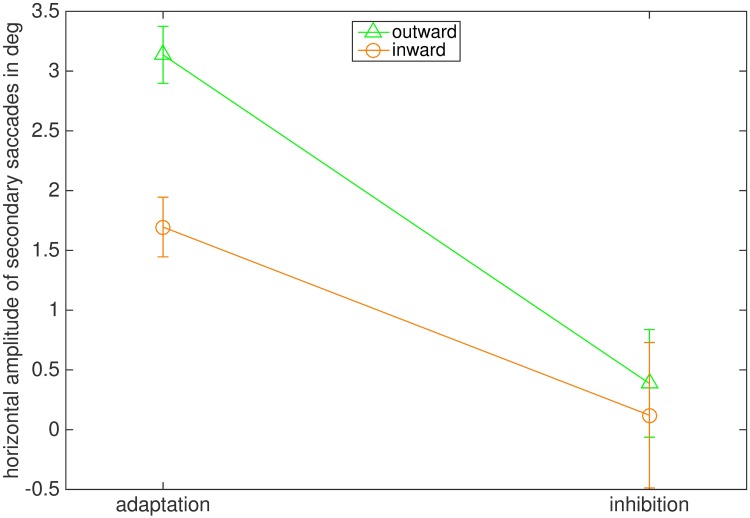
Mean horizontal amplitude of secondary saccades in degree. Positive values indicate secondary saccade direction toward the stepped target position, while negative values indicate that the secondary saccades were made toward the initial target position. The bars indicate the standard error of the mean.

In normal adaptation, secondary saccades are usually corrective and directed toward the post-saccadic location of the stepped target. In our inhibition conditions, however, participants were instructed to direct their gaze to the initial position of the target and avoid looking at the stepped target. Therefore, we wondered if secondary saccades moved gaze closer to the stepped target, which is the case for normal corrective saccades or toward the original landing position of the target. For this analysis we selected secondary saccades from all trials in which the primary saccades landed between the initial and the stepped target position (Fig 4c and 4d). We then calculated the difference between the horizontal landing position of the primary saccade and the horizontal landing position of the secondary saccade. Since target steps were in opposite directions in the outward and inward conditions we flipped the sign of the saccade direction in the outward condition such that a positive value always corresponded to a secondary saccade towards the stepped target while a negative value indicated a secondary saccade back to the initial target position. We then calculated the average of the horizontal position change for each condition. Fig 6 shows the results. As expected, in the adaptation conditions the secondary saccades were, on average, made toward the stepped target position. In the inhibition conditions, the average was close to zero, indicating that, on average, secondary saccades did not move gaze closer to the stepped target. The ANOVA reported a significant main effect of the instruction (F(1,7) = 37.050, p < .001, η2 = .861). The main effect of adaptation direction (F(1,7) = 2.911, p = .139) and the interaction between instruction and adaptation direction (F(1,7) = 1.188, p = .318) were not significant.

#### Latencies

**Fig 7:**
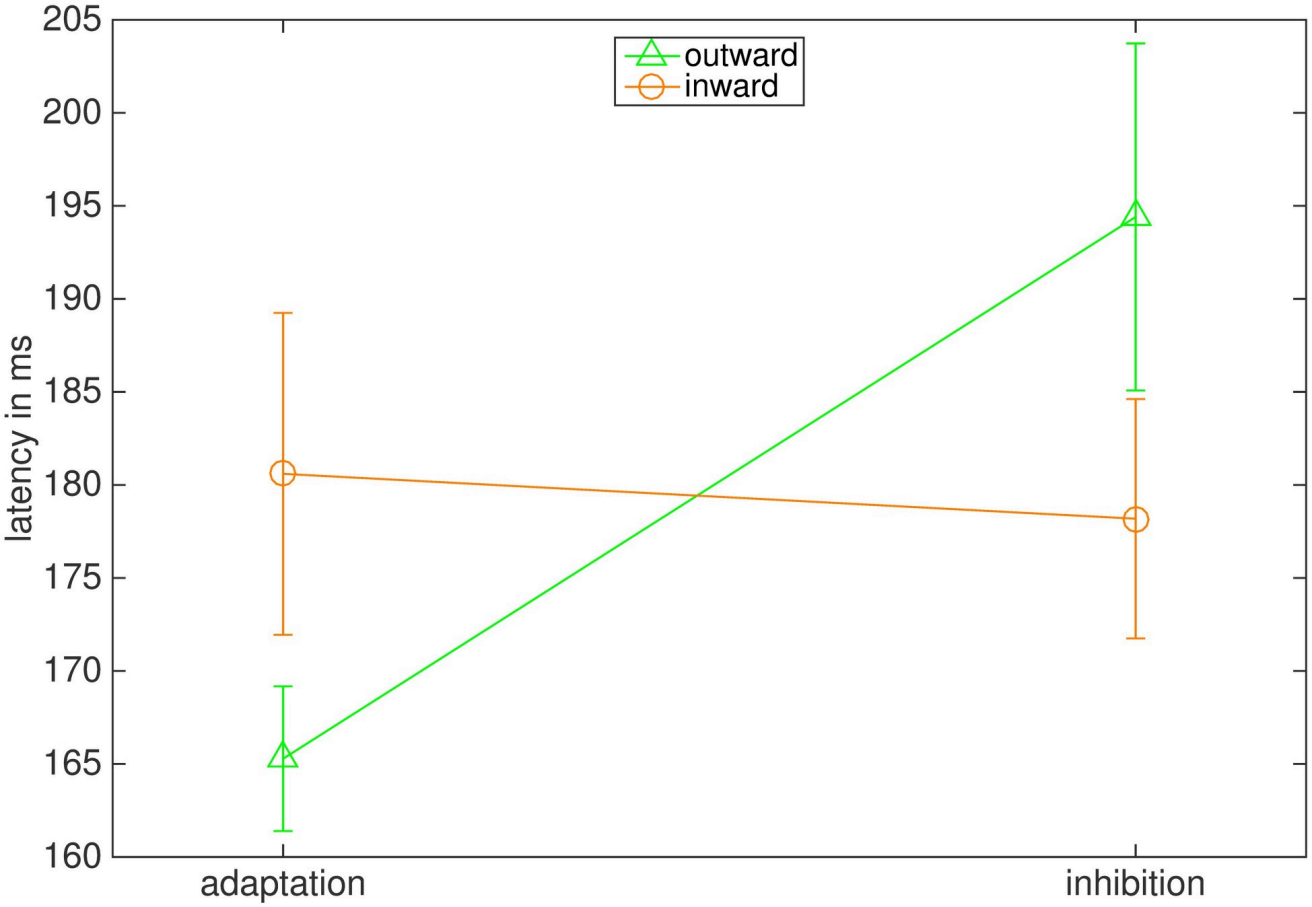
Mean latencies in ms. The bars represent the standard error of the mean.

We also analyzed the influence of adaptation direction and instruction on saccadic latency (Fig 7). The ANOVA reported a significant main effect of instruction (F(1,7) = 6.018, p = .044, η2 = .462) and a significant interaction between instruction and adaptation direction (F(1,7) = 10.944, p = .013, η2 = .610). The main effect of adaptation direction was not significant (F(1,7) = 0.030, p = .868). Latency was 194.40 ms (26.38 ms) in the outward inhibition condition and significantly larger than in the outward adaptation condition (165.28 ms (11.00 ms), t = 3.494, p = .020). Latencies in the inward inhibition 178.18 ms (18.20 ms) and the inward adaptation condition did not differ significantly from each other (180.60.69 ms (24.48 ms), t = -0.407, p = .697).

**Fig 8:**
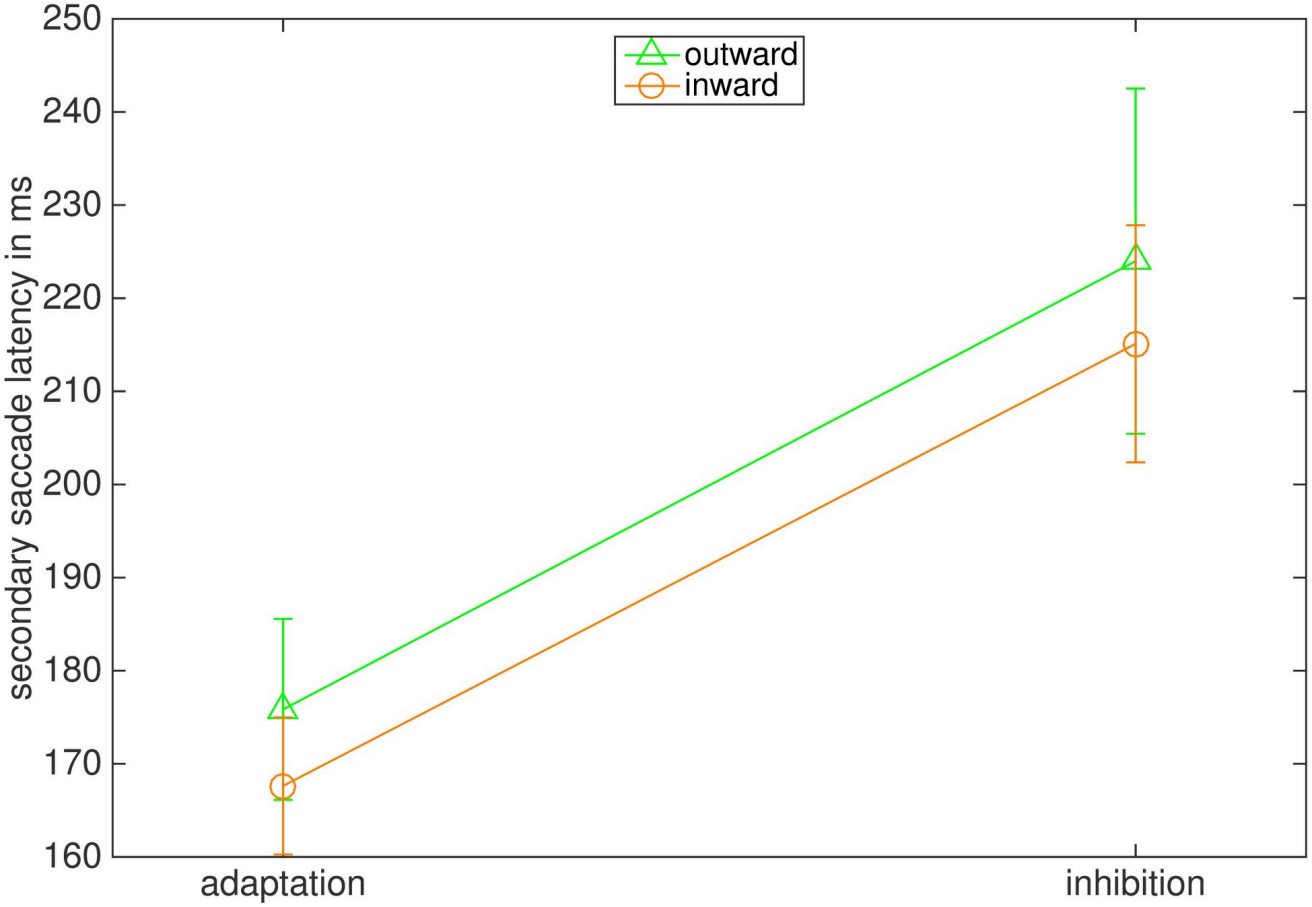
Mean latencies of secondary saccades in ms. The bars represent the standard error of the mean.

The latencies of secondary saccades are depicted in Fig 8. As secondary saccades are not primed by the appearance of a target, their latency is computed by the time interval between primary saccade’s offset and secondary saccade’s onset. The ANOVA reported a significant effect of the instruction (F(1,7) = 7.55 p = .029, η2 = .519). The interaction between instruction and adaptation direction (F(1,7) = 0.001, p = .970) and the main effect of adaptation direction were not significant (F(1,7) = 2.136, p = .187). The average latency of secondary saccades was 223.98 ms (87.06 ms) in the outward inhibition and 175.85 ms (68.03 ms) in the outward adaptation condition. In the inward inhibition condition latency was 215.10 ms (88.37 ms) and 167.63 (61.60 ms) in the inward adaptation condition.

## Experiment 2

The results of Experiment 1 showed that the instruction to continue to look at the initial position of the target led to a smaller change in saccadic gain than the instruction to follow the target to its final position. Saccadic latency, frequency, and direction of secondary saccades were also influenced by instruction type. However, the effects were somewhat different for inward and outward adaptation. Specifically, inhibition of inward adaptation still produced some residual adaptation, which was not the case for inhibition of outward adaptation. In Experiment 2 we sought to replicate the findings with smaller step sizes. While a step size of 40% of the primary saccade amplitude is a common size for saccadic adaptation experiments we wondered whether smaller step sizes, that may have a lesser chance of being consciously perceived, would lead to different efficacy of inhibition. Thus we shortened the target steps in the second experiment in order to reduce any conscious visual perception of the step during the experiment. Again, participants were aware—from the instruction- that they either had to look at the first target location or follow the target to its final location, but if intra-saccadic target steps are small enough they are often imperceptible and participants might not perceive a difference between the two locations.

### Method

#### Sample

The sample consisted of eight participants (5 male, 3 female) aged between 23 and 53 (M = 30.75, SD = 9.90). All participants were right handed and had normal or corrected-to-normal vision. The participants were recruited from the Department of Psychology and gave informed consent concerning the collection and analysis of the data in written form. Six of the participants had previously taken part in Experiment 1. The delay between participation in the first and second experiment was 3.5 months.

#### Experimental setup and design

Setup, Design and Data Analysis were identical to Experiment 1. The only difference was the size of the intra-saccadic target step, which was 15, 20 or 25 percent of the primary saccade amplitude.

### Results

#### Gain change

**Fig 9:**
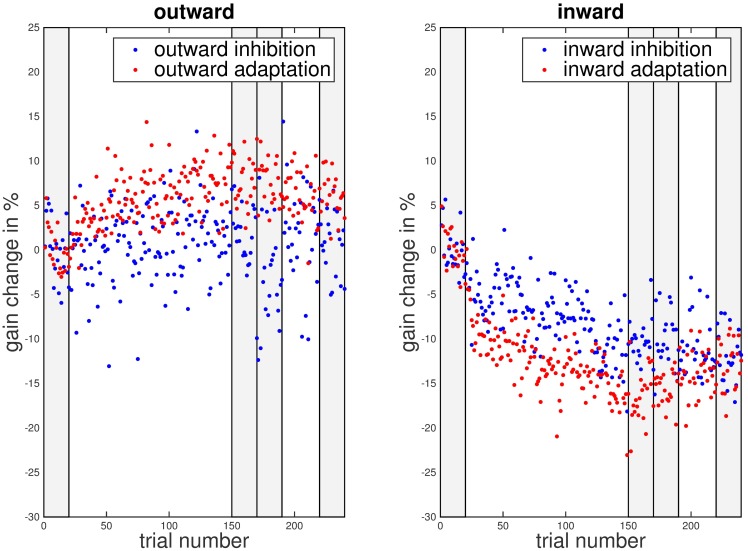
Gain change over trial number for different instruction types and adaptation directions in Experiment 2. Data from the inhibition conditions is marked by blue dots, data from the adaptation condition by red dots. The shaded areas mark the phases used for gain change analysis: pre-adaptation (trials 1–20), late adaptation (trials 151–170), post-adaptation (trials 171–190) and late post-adaptation (trials 221–240).

As in the first experiment, the saccadic gain change followed the typical exponential learning curve in the outward adaptation condition. In the outward inhibition condition gain change remained around zero throughout the experiment. In the inward conditions however, saccadic gain decreased roughly in parallel in both conditions. As in Experiment 1, the gain change was stronger in the adaptation than in the inhibition condition (Fig 9).

**Fig 10:**
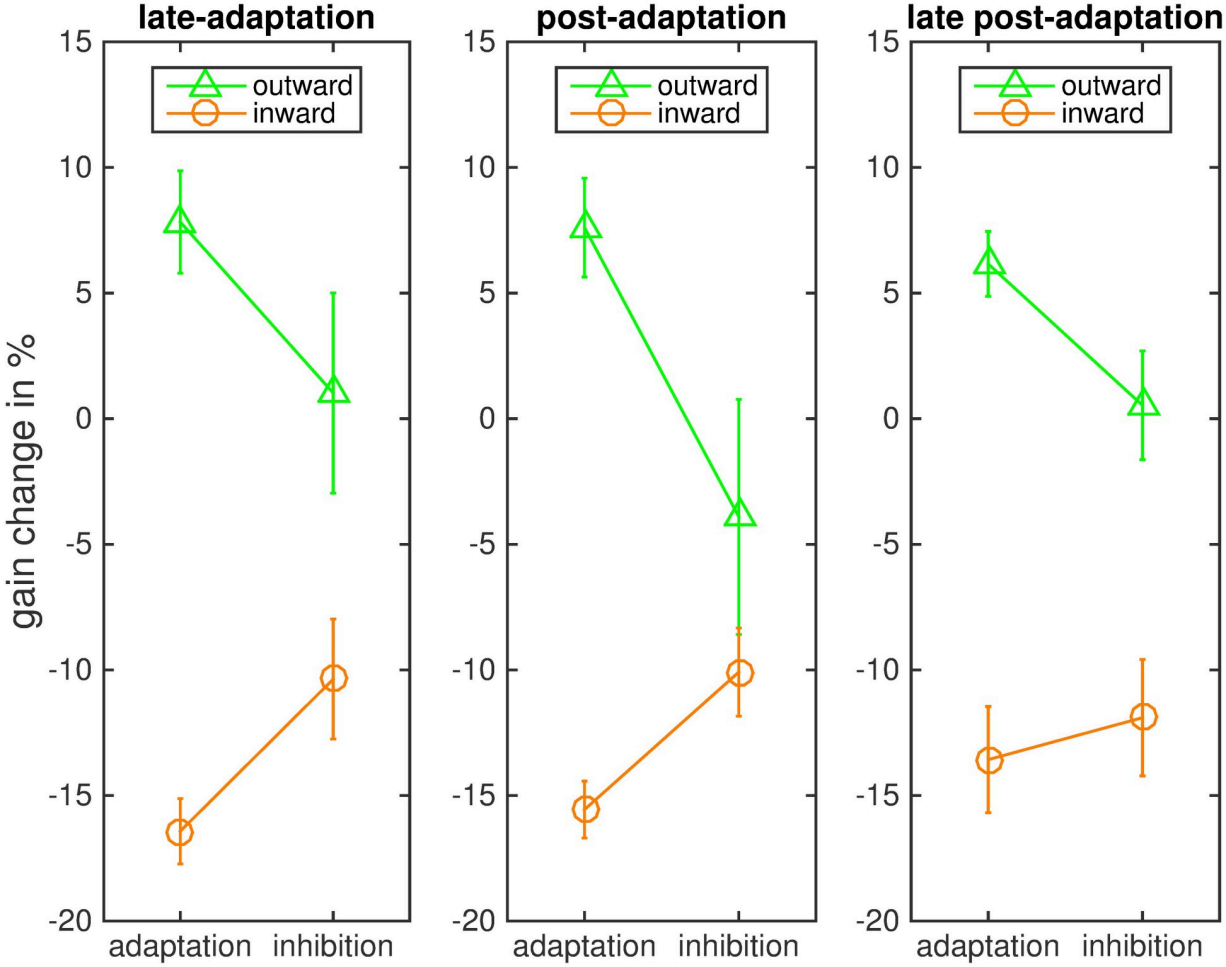
Mean saccadic gain in the four conditions for each of the three phases. The bars indicate the standard error of the mean.

We conducted ANOVAs for the mean gain change in the last 20 adaptation trials, the first 20 post-adaptation trials, and the last 20 post-adaptation trials. The ANOVA of the last 20 adaptation trials showed the expected significant main effect for the adaptation direction (F(1,7) = 65.074, p < .001, η2 = .903) and the expected lack of a main effect of instruction (F(1,7) = 0.028, p = .872). The interaction of instruction and adaptation direction was significant (F(1,7) = 6.299, p = .040, η2 = .474). The same pattern of results was found for the post-adaptation trials: The main effect of adaptation direction was significant (F(1,7) = 41.030, p < .001, η2 = .854) as was the interaction of adaptation direction and instruction (F(1,7) = 11.824, p = .011, η2 = .628). The main effect of instruction was not significant (F(1,7) = 1.020, p = .346). In the last 20 post-adaptation trials, the main effect of adaptation direction remained significant (F = 678.246, p < .001, η2 = .990), but the interaction of adaptation direction and instruction was not significant (F = 2.235, p = .179). The main effect of instruction was also not significant (F = 1.055, p = .338). Values are depicted in Fig 10.

The average gain change (and standard deviation) in the late adaptation trials was 7.83% (5.77%) for outward adaptation, 1.02% (11.27%) for outward inhibition, -16.42% (3.68%) for inward adaptation and -10.37% (6.76%) for inward inhibition. In the post-adaptation trials, mean gain change was 7.6% (6.8%) for outward adaptation, -3.9% (10.5%) for outward inhibition, -15.6% (6.8%) for inward adaptation and -10.1% (6.9%) for inward inhibition. In the last post-adaptation trials mean gain change was 6.16% (3.66%) for outward adaptation, 0.53% (6.12%) for outward inhibition, -13.57% (5.98%) for inward adaptation and -11.90% (6.55%) for inward inhibition.

The gain change in the last 20 adaptation trials deviated from zero in the inward adaptation (t = 12.612, p < .001), the inward inhibition (t = -4.340, p = .003) and the outward adaptation condition (t = 3.839, p = .006), but not in the outward inhibition condition (t = 0.2569, p = .805). The same was true for the post-adaptation trials: Gain change deviated from zero in the inward adaptation (t = -13.76, p < .001), the inward inhibition (t = -5.742, p = .002), and the outward adaptation condition (t = 3.866, p = .012), but not in the outward inhibition condition (t = -0.835, p = .431). For the last 20 post-adaptation trials, gain change also deviated from zero in the inward adaptation (t = -6.422, p = < .001), the inward inhibition (t = -5.140, p = .001) and the outward adaptation condition (t = 4.765, p = .002). In the outward inhibition condition gain change did not differ from zero (t = 0.246, p = .813) Hence, like in Experiment 1, the instruction to inhibit inward adaptation led to some residual adaptation, while inhibition of outward adaptation prevented a significant change of saccadic gain during the experiment. However, unlike in Experiment 1, the adaptation remained even in the last post-adaptation trials for inward steps, both for the instruction to inhibit and the instruction to adapt, and for outward steps with the instruction to adapt.

#### Secondary saccades

**Fig 11:**
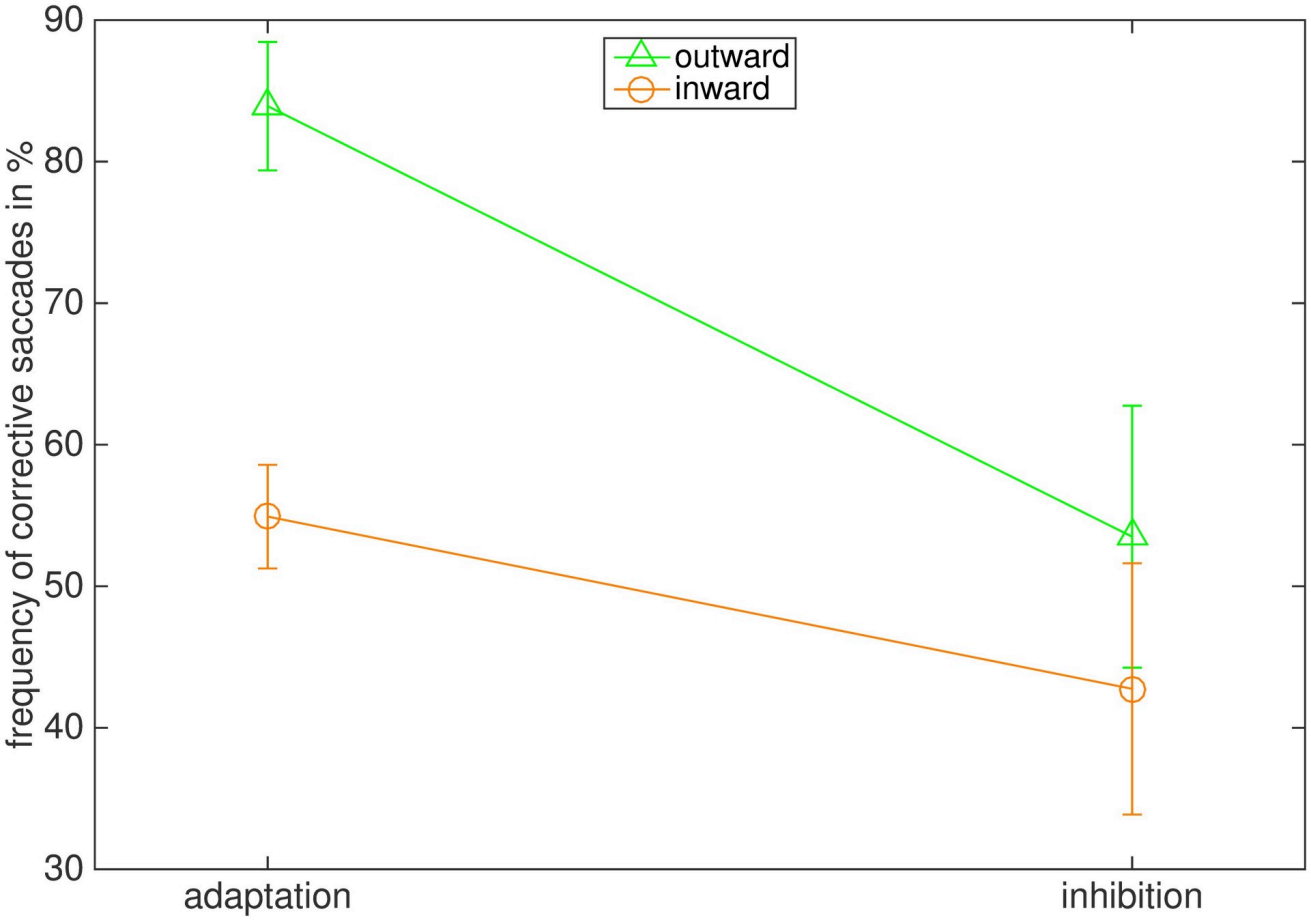
Average frequency of corrective saccades in the four conditions. The bars indicate the standard error of the mean.

Fig 11 illustrates the effect of instruction and adaptation direction on the frequency of corrective saccades towards the final target location. As in Experiment 1, more corrective saccades were executed in the adaptation than in the inhibition conditions, and more corrective saccades were performed when the target stepped outward. The average frequency of corrective saccades was 53.5% (26.2%) for outward inhibition and 84% (12.8%) for outward adaptation. In the inward inhibition condition the average frequency was 42.8% (25.1%) and 54.9% (10.4%) for inward adaptation. The ANOVA supported the results, reporting a significant main effect of instruction (F(1,7) = 11.024, p = .013, η2 = .612) and adaptation direction (F(1,7) = 9.515, p = .018, η2 = .576). The interaction of instruction and adaptation direction was not significant (F(1,7) = 4.567, p = .070).

Similar to Experiment 1, GLMs of the frequency of corrective saccades with the predictor trial number showed a significant decrease in the inward adaptation condition (ß^ = -0.006, t = -2.440, p = .016) but not in any of the other conditions (outward inhibition (ß^ = -0.004, t = -1.9228, p = .056), outward adaptation (ß^ = -0.003, t = -1.788, p = .076), inward inhibition (ß^ = .002, t = 0.794, p = .428).

**Fig 12:**
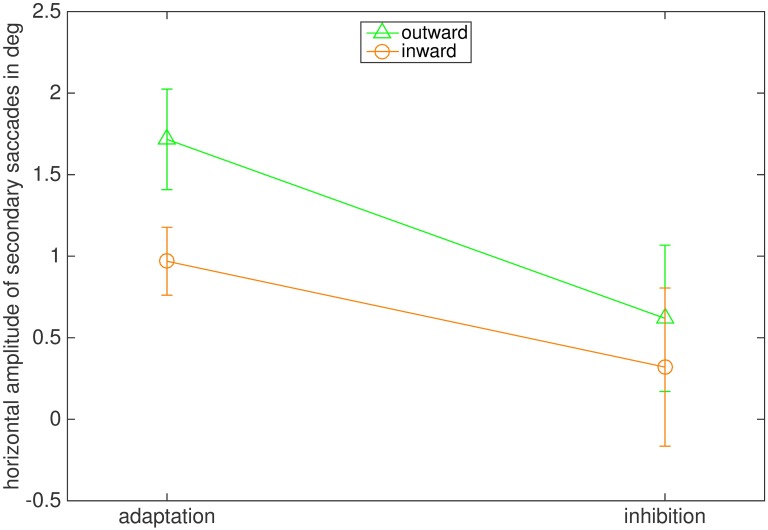
Mean horizontal amplitude of secondary saccades in degree. Positive values indicate that on average secondary saccades were made toward the stepped target position, while negative values indicate that the secondary saccades were made toward the original target position. The green line marks the outward condition, the orange line stands for the inward condition. The bars indicate the standard error of the mean.

Results for the direction of secondary saccades were also very similar to those of Experiment 1 (Fig 12). In both adaptation conditions secondary saccades went on average toward the stepped target, while there was no preference for neither the original target position nor the position of the stepped target in the inhibition conditions. These results were confirmed by the ANOVA, reporting a significant main effect of the instruction (F(1,7) = 7.402, p = .030, η2 = .514). The main effect of adaptation direction (F(1,7) = 1.172, p = .315) and the interaction of adaptation direction and instruction (F(1,7) = 0.588, p = .468) were not significant.

#### Latencies

**Fig 13:**
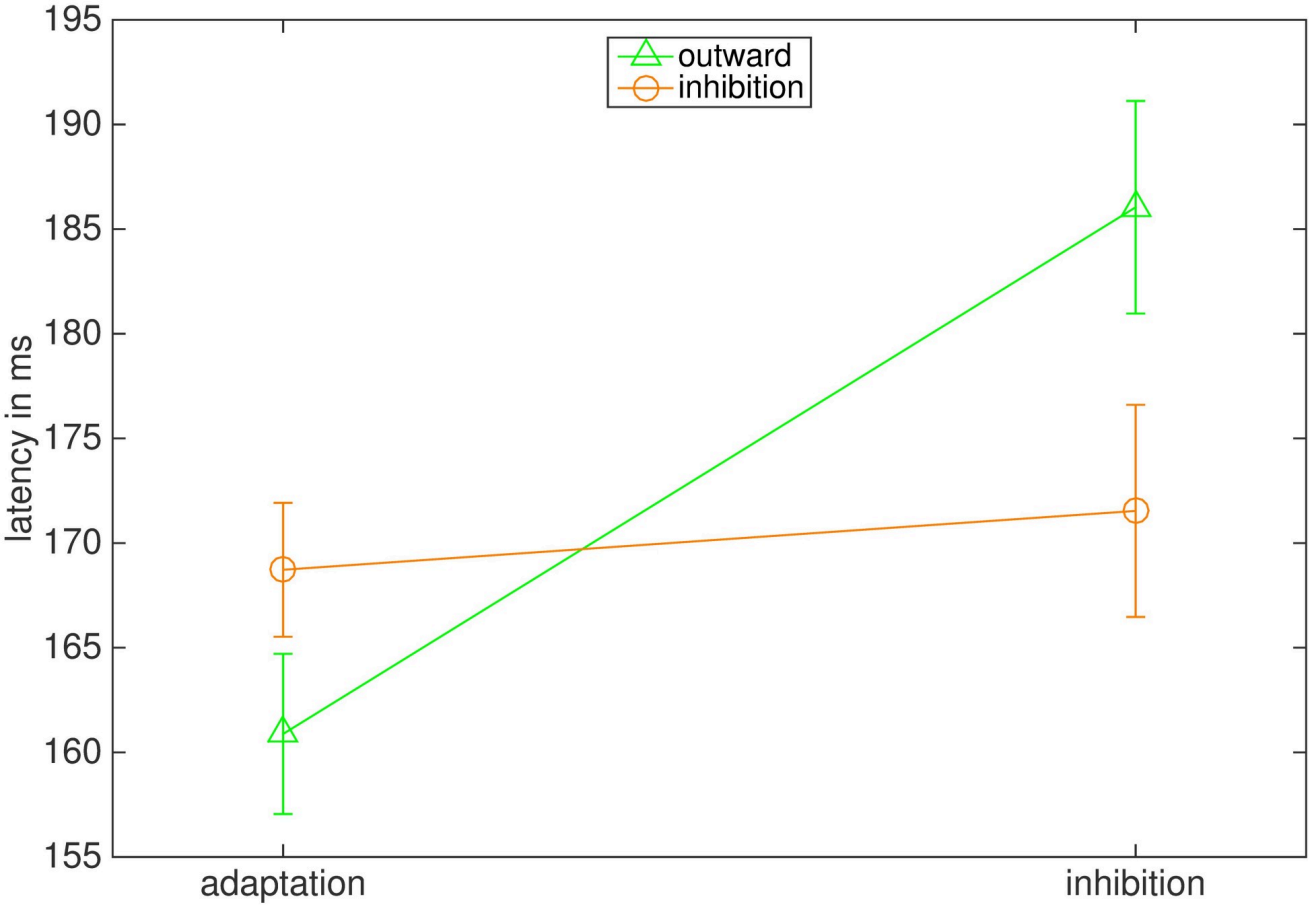
Mean latencies in ms. The bars represent the standard error of the mean.

As in Experiment 1, latencies in the inward conditions were barely influenced by the instruction, while latencies in the outward condition showed a large influence of the instruction (Fig 13). Accordingly, the ANOVA reported a significant main effect of instruction (F(1,7) = 13.295, p = .008, η2 = .655) and also a significant interaction of instruction and adaptation direction (F(1,7) = 16.325, p = .005, η2 = .700). The main effect of the adaptation direction was not significant (F(1,7) = 1.411, p = .274). The average latency in the outward inhibition condition (186.04 ms (14.36 ms)) was significantly greater than in the outward adaptation condition (160.04 ms (10.83 ms), t = 4.436, p = .006), while latencies in the inward inhibition (171.54 ms (14.33 ms)) and the inward adaptation condition (168.73 ms (9.05 ms)) did not differ significantly from each other (t = 0.793, p = .454).

**Fig 14:**
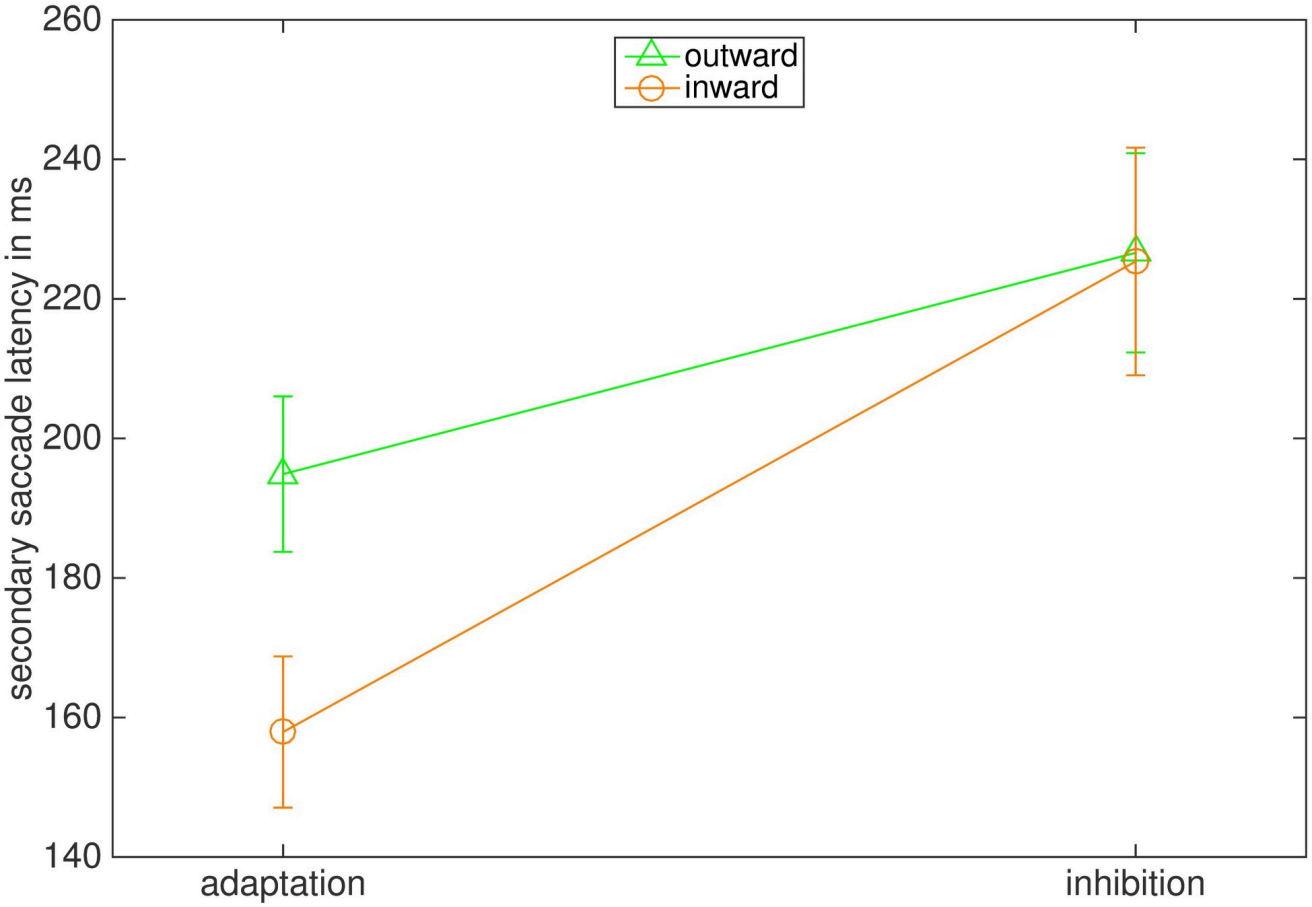
Mean latencies of secondary saccades in ms. The bars represent the standard error of the mean.

The latencies of secondary saccades were also influenced by the instruction (Fig 14). The ANOVA reported a significant main effect of instruction (F(1,7) = 44.367, p < .001, η2 = .864). The interaction between instruction and adaptation direction (F(1,7) = 2.547, p = .155) and the main effect of adaptation direction were not significant (F(1,7) = 3.853, p = .090). In the outward inhibition condition the average latency of secondary saccades was 226.61 ms (78.31 ms) and in the outward adaptation condition 194.88 ms (73.14 ms). In the inward condition latencies were 225.36 ms (78.87 ms) in the inhibition condition and in the adaptation condition 157.92 ms (68.84 ms). Hence, latencies of secondary saccades increased for both adaptation directions when participants were told to inhibit amplitude adjustment.

## Discussion

The data of both experiments consistently showed the same pattern of results: First, voluntary inhibition of adaptation leads to lower gain change during double-step trials than regular adaptation. Second, some residual adaptation is observed despite voluntary inhibition in inward but not outward adaptation. Third, latencies of the primary saccades are increased when adaptation is inhibited in the outward condition. Fourth, latencies of the secondary saccades are increased in the inhibition condition for both directions.

The first result implies that the type of instruction influences saccadic adaptation. Consequently, volitional control can be exerted on the adjustment of saccadic amplitude. Previous studies suggested that any volitional aspect in saccadic adaptation is rather small [21,19,18]. However, our results are consistent with the findings of Kowler and Blaser [23], who reported that participants were able to execute more precise saccades and to reduce the amount of secondary saccades after being instructed to make an accurate eye movement toward a target. Like in their study, our participants decreased their number of secondary saccades when they were instructed to remain fixated at the initial target position.

The second result showed that inhibition of amplitude adaptation was only completely successful in the outward conditions. Outward adaptation is known to be more difficult, slower and weaker than inward adaptation [14,15,16]. In the inward inhibition conditions, the change in saccadic gain differed from zero, accordingly adaptation could not be fully suppressed. This is further evidence for the idea that different mechanisms underlie gain increase and gain decrease [14,15,16,11,26,12]. Since the attempt to inhibit saccadic adaptation due to instruction requires volitional, active control and was more successful in the outward condition, our findings support the suggestion that outward adaptation is a rather active process, while inward adaptation is thought to involve an uncompensated fatigue effect, which cannot be fully controlled [11,27,12]. Hence, when interpreting the results of this study, one should take the natural hypometria of the saccadic system into account. As saccades tend to undershoot the target, the successful inhibition of adaptation of saccadic amplitude in the outward condition could also be partly due to the natural facilitation of inward adaptation in contrast to outward adaptation. In addition, one should keep in mind that outward adaptation is generally weaker and slower than inward adaptation and some residual adaptation in the outward inhibition condition might only appear after a larger number of trials.

The difference between inward and outward adaptation is also seen in the third result. In the outward condition participants needed more time to initiate a saccade when they had been told to suppress adaptation. This was not the case in the inward condition. Apparently, some process involved in the inhibition of outward adaptation is active before saccade initiation and needs time to complete. The increase of latency hence might reflect the influence of instruction on target remapping in advance of the execution of the saccade, being more or even exclusively active in outward adaptation [11,12]. Latency increases of secondary saccades in the inhibition condition might link to results of studies with anti-saccades, where subjects are instructed to make an intentional saccade in the opposite direction of an appearing target [28]. Latencies of anti-saccades are longer than those of reflexive saccades [29], which can be traced back to two processes involved in the generation of anti-saccades: the volitional suppression of the reflexive saccade toward the target [28,30] and the adjustment of the vector specifying direction and amplitude of the anti-saccade [31]. The increase in latency for secondary saccades following the instruction to inhibit adaptation suggests that avoiding to look at the stepped target by either suppressing the reflexive secondary saccade or guiding it in the opposite direction is based on the same mechanisms as anti-saccades.

The inhibition of outward adaptation in our study might be another example of successful inhibition of a reflexive saccade combined with vector adjustment prior to saccade execution.

The reduction of adaptation in the inhibition conditions was found at the end of the adaptation phase (last 20 adaptation trials) and also in the first 20 post-adaptation trials. Moreover, it was also significant in the last 20 post-adaptation trials in Experiment 1, and partially in Experiment 2. However, in Experiment 2 outward adaptation in the last 20 post-adaptation trials was not significantly different from zero, i.e., it had decayed during the target-off post-adaptation trials. The decay may be linked to the smaller step size in Experiment 2. Still, significant adaptation remained even in the last post-adaptation trials for inward steps, both for the instruction to inhibit and the instruction to adapt, and for outward steps with the instruction to adapt. Hence saccadic gain seems to be quite stable.

In conclusion, results of the current study show that adaptation of saccadic amplitude is clearly influenced by the instruction to inhibit or adapt, as gain change was smaller in the inhibition than in the adaptation conditions. As the instruction to inhibit adaptation was only completely successful for outward adaptation, alterations to the remapping of the target position might be the key aspect to inhibition of saccadic amplitude.
